# Defining the Parameters of Incidental Learning on a Serial Reaction Time (SRT) Task: Do Conscious Rules Apply? 

**DOI:** 10.3390/brainsci2040769

**Published:** 2012-12-17

**Authors:** Lynne A. Barker

**Affiliations:** Brain, Behaviour and Cognition Group, Department of Psychology, Sheffield Hallam University, Collegiate Crescent Campus, Sheffield, S10 2BP, UK; E-Mail: l.barker@shu.ac.uk; Tel.: +44-0-114-225-5379; Fax: +44-0-114-225-2430

**Keywords:** incidental, implicit, Serial Reaction Time (SRT), explicit, learning

## Abstract

There is ongoing debate about the contribution of explicit processes to incidental learning, particularly attention, working memory and control mechanisms. Studies generally measure explicit process contributions to incidental learning by comparing dual- to single-task sequence learning on some variant of a Serial Reaction Time (SRT), usually adopting an auditory tone counting task as the secondary task/memory load. Few studies have used secondary working memory stimuli with the SRT task, those that have typically presented secondary stimuli, before, after or between primary task stimuli. Arguably, this design is problematic because participants may potentially “switch” attention between sequential stimulus sources limiting the potential of both tasks to simultaneously index shared cognitive resources. In the present study secondary Visual and Verbal, memory tasks were temporally synchronous and spatially embedded with the primary SRT task for Visual and Verbal dual-task conditions and temporally synchronous but spatially displaced for Visual-Spatial and Verbal-Spatial Above/Below conditions, to investigate modality specific contributions of visual, verbal and spatial memory to incidental and explicit sequence learning. Incidental learning scores were not different as an effect of condition but explicit scores were. Explicit scores significantly and incrementally diminished from the Single-task through Visual-Spatial Below conditions; percentage accuracy scores on secondary tasks followed a significant corresponding pattern suggesting an explicit learning/secondary memory task trade-off as memory demands of tasks increased across condition. Incidental learning boundary conditions are unlikely to substantially comprise working memory processes.

## 1. Introduction

Incidental learning is broadly defined as the acquisition of information (sequences, patterns, motor programmes or other regularities) without conscious or explicit awareness of the process of learning or explicit knowledge of the information acquired [1,2]. Incidental learning (sometimes termed procedural or implicit) is a ubiquitous foundational cognitive ability thought to support diverse complex functions, including processing and production of language, musical and arithmetical ability, non-verbal social cue encoding and decoding, behavioural insight, acquisition of constructs, and myriad motor skills from juggling to hopscotch [3,4,5,6,7]. Explicit learning by contrast is typically amenable to verbal description, accompanied by awareness of the process (the learning episode) and the “product” (the information learned).

While there is general agreement about the ubiquitous nature of incidental learning there is debate about the contribution of explicit processes [8,9,10], specifically the role of attention and working memory, to incidental learning. Some researchers have proposed that working memory and attention resources are crucial to incidental learning [11,12,13,14], others have suggested minimal contribution [15,16,17,18], or that learning proceeds entirely automatically [19]. 

Hsiao and Reber [20] observed that most models of incidental sequence learning incorporate (usually tacitly), a “limited-capacity short-term memory system” (p. 341). The relative contribution of memory processes to incidental learning is typically investigated by either comparing working memory capacity with learning score, comparing learning performance of high and low memory span groups, or by comparing learning on an incidental learning task with no secondary load (single-task design) to learning with the presence of secondary memory/attention tasks (dual-task design). Methods derive from the assumption that working memory capacity constrains learning on tasks that load the same cognitive processes; working memory capacity should either correlate with incidental learning score, and/or secondary tasks should load working memory and diminish incidental learning score, if completion of both tasks depends upon the same cognitive processes. The standard approach utilises some variant of a standard Serial Reaction Time (SRT) task to measure incidental sequence learning and often includes a concurrent auditory tone-counting task as the secondary memory task/attentional load [1,11,14,21,22,23,24,25]. 

In the canonical version of the SRT task visual stimuli follow a repeating sequence and participants respond to the position of the target stimulus on screen by pressing a corresponding keypad as quickly as possible [1,11]. Reaction time (RT) responses are recorded and learning is shown by faster responses to sequence compared to non-sequenced stimulus learning trials. Early research suggested that the effect is robust across single tasks but may be diminished or abolished entirely with the introduction of a secondary concurrent tone counting task [11,23]. Effects of secondary tasks on incidental learning performance are variously thought to occur due to a processing bottleneck when separate motor responses are required for both tasks, interference effects in working memory, central resource (attention) capacity constraints or temporally asynchronous dual-task stimuli, depending upon theoretical position and experimental design [11,17,21,22,23,24,25,26]. Additionally, there is huge variability across dual-task design in different studies. In many studies secondary stimuli are embedded in some way within the primary task (either temporally or visually depending on the secondary task) and occur either before or after sequence trials (the convention with secondary memory tasks), or between Stimulus-Response of sequence trials (secondary auditory tone counting tasks), with few exceptions [10]. However, in designs where secondary stimuli are not synchronous with primary stimuli it is possible for participants to switch attention between two sequential task stimuli rather than share memory and attentional resources across tasks. Synchronicity, on the basis of the current study and other work [10], refers to dual-task designs whereby secondary task stimuli constitute both a primary sequence task trial and crucial secondary task target stimuli. This design is more likely to require allocation of shared resources across tasks if both tasks recruit the same cognitive processes. 

What remains to be determined is whether key component functions of incidental learning are implicit/explicit, visual/spatial or a combination of visual and spatial and whether such functions load working memory resources since most dual-task designs have used auditory tone counting rather than working memory secondary task stimuli. This knowledge may lead to a better understanding of the underlying cognitive processes responsible for incidental learning and provide important insights into the organization of cognitive architecture governing complex behaviour.

Barker and Andrade [27] proposed that incidental learning occurs due to an associative learning mechanism that automatically detects environmental co-variations resulting in processing fluency for sequential patterns and/or reliably co-varying stimuli. This hypothesis tacitly assumes a negligible role of working memory to incidental learning although studies have yielded mixed results. Frensch and Miner made similar assumptions but proposed a role of short-term memory for encoding sequence elements [19]. However, Unsworth and Engle [28] found that working memory span (high *versus* low) was uncorrelated with incidental sequence learning and positively correlated with explicit sequence learning, suggesting that incidental learning does not depend upon working memory processes. 

Song, Marks, Howard and Howard [29] tested healthy older adults on a cued variant of a probabilistic SRT task to distinguish effects of explicit learning on incidental learning from effects on motor performance. The authors argued that reported variability in incidental learning ability in groups with reduced memory capacity (stroke patients and healthy elderly [30,31]) reflected effects of explicit learning, rather than diminished incidental learning, on motor performance. Findings showed that incidental sequence learning was not influenced by concurrent explicit learning in older adults, and was not correlated with working memory span, although explicit learning did negatively impact motor performance. Similarly, Feldman, Kerr and Streissguth found that incidental learning on an SRT task was not significantly correlated with reasoning, processing speed and working memory tasks, whilst explicit learning measured by a generation task correlated reliably with most measures [32]. Finally, Remillard [18] reported that the incidental sequence learning mechanism operates over a range of at least seven sequence elements exceeding the purported capacity of explicit spatial working memory. Remillard concluded that incidental sequence learning is not bound by the capacity limits of working memory [18]. 

In contrast, Bo, Jennet and Seidler [12] found that visual and verbal working memory capacity correlated with rate of reaction time change on a SRT task suggesting a relationship between working memory and incidental sequence learning. They also found that visuospatial working memory ability explained a significant portion of the variance in rate of SRT performance change across individual participants. The authors concluded that working memory processes make a key contribution to incidental learning on SRT tasks. Similarly, Barker *et al.* found that some patients with frontal brain injury (the region thought to subserve working memory) and visual memory deficits were impaired on an incidental SRT task compared to matched controls [2]. However findings were not clear-cut as patients with intact visual memory (but other cognitive deficits) also showed diminished incidental learning compared to controls hinting that functions separate from visual memory also contribute to incidental learning performance. 

Hsiao and Reber [20] manipulated RSOA (response-secondary stimulus onset asynchrony) in a series of dual-task experiments. The authors posited that any account of the varying impact of RSOA on sequence learning must include a working memory component that sequentially codes stimulus representations in a time-locked manner, so that incoming stimulus items will override previous representations if they overlap and so compromise sequence learning. Frensch, Lin and Buchner [24] similarly proposed that dual-task interference effects occur because secondary tasks disrupt the availability of successive sequence information through “interference mechanisms” in short-term memory based on findings that learning is diminished when secondary task inter-stimulus intervals increase. There is also some evidence of an interaction between age, incidental learning and memory/attentional resources with a secondary task load impeding incidental learning in an elderly group compared to a younger group thought to occur due to age-related attenuation of working memory capacity [33]. 

Other researchers investigating the putative contribution of working memory processes to SRT learning have adapted the standard dual-task approach (secondary task = an auditory tone counting task), by embedding visual and verbal memory task stimuli before, after or between primary SRT task stimuli. Stadler [16] presented participants with a series of letters (5, 7 or 9) for a duration of 5 s preceding a SRT task and found that memory load (letters accurately recalled after RT trials) did not impede sequence learning. Heuer and Schmidtke [25] presented Brooks visuo-spatial or Brooks verbal task stimuli [34] before SRT task stimuli; items were recalled 90 s later after the SRT task block was completed. They found no interference effects of secondary memory tasks on learning or the expression of learning, although a secondary auditory go/no go task did impede sequence learning. The authors concluded that secondary task interference is not capacity-based but occurred for the go/no go condition via disruption of contiguous elements of the sequence. These studies devised dual-task designs whereby secondary memory stimuli were not synchronous with primary SRT task stimuli, although they did require representations to be maintained in memory during performance of the sequence task. In addition, both studies reported almost 100% accuracy scores on the secondary tasks suggesting possible ceiling effects.

On the basis of earlier findings of secondary task effects (auditory tone counting, “go-no go”), and no effect of secondary (embedded memory) tasks on SRT learning, Schmidtke and Heuer [35] predicted that synchronous primary and secondary task stimuli should result in unimpeded learning on both tasks. They concluded that task integration and task-relevance of secondary stimuli was a critical component of whether secondary stimuli became integrated with the primary task to produce unimpeded learning. The authors found that when the secondary task consisted of a random series of tones sequence learning was impeded whilst a repeated sequence of tones that contained the same number of elements as the visual task resulted in higher learning scores than for the single-task alone. They concluded that task integration accounted for these data and that the task-relevance of secondary stimuli was a critical component of whether secondary stimuli became integrated. When tones were presented without instruction the secondary sequence was only minimally learned suggesting that mechanisms of implicit learning are not wholly non-attentional. 

Schumacher and Schwarb [28] investigated the effects of secondary task load on incidental sequence learning in an experiment where the secondary tone identification task was presented concurrently (occurred simultaneously with the visual SRT task stimulus), or occurred after a brief stimulus onset asynchrony (SOA) interval. Surprisingly, they found impaired dual-task learning in the first condition but not the second and concluded that simultaneous dual-task processing that requires response selection disrupts sequence learning as opposed to attentional switching between two tasks. Of note, the authors proposed that secondary task stimuli were “synchronous” with primary task stimuli in these experiments because they co-occurred with visual sequence trials. Secondary task stimuli were not synchronous in the sense that they simultaneously represented a sequence trial maintaining the temporal integrity of the sequence, and also represented secondary task target stimuli. Similarly, secondary task-stimuli were presented aurally not visually as with primary visual SRT task stimuli, and presentation rate varied to correlate or not with the primary SRT task. So it is possible that secondary task effects on sequence learning might occur due to perceptual integration errors between incoming visual and auditory stimuli rather than as a result of competing response selection or shared limited capacity resources across tasks. Importantly, Schumacher and Schwarb [28] reported that 15 of 21 dual-task SRT studies utilising auditory tone counting as the secondary task showed dual-task learning with only six studies showing dual-task interference effects. Thus, the weight of evidence suggests that SRT task learning is not impeded by a secondary auditory tone counting task, or secondary memory tasks that are embedded within, but not synchronous with primary SRT task stimuli. There is variability however across studies between presentation rates, mode of instruction, inter-trial interval, and modality of secondary task that might somewhat account for contrasting findings.

Other researchers have developed dual-task designs whereby secondary task stimuli appear as targets for reaction time responses in the primary incidental task, and simultaneously constitute target stimuli for the secondary learning or memory task [10]. Jiménez and Méndez [10] assessed the differential effects of incidental and explicit “shape” learning on a probabilistic (grammar learning) SRT task using synchronised secondary stimuli. The authors relied upon the same four stimuli to specify the location of the sequence trials, to predict the next position of the sequence (on a shape learning task) and to comprise the secondary counting task. Findings showed that incidental learning was acquired and expressed regardless of the influence of explicit knowledge. However, secondary tasks were not designed to access specific verbal and visual working memory resources although explicit shape learning did require visual processing concurrent with incidental SRT task learning. In other work, Jiménez and Vázquez [17] reported that interference effects of secondary tasks diminished when a secondary auditory tone task co-varied with the location of primary task stimuli. These findings suggest that when secondary tasks are synchronous with [10], or reliably co-vary with the primary task, learning on incidental and explicit tasks may proceed undiminished in line with Barker and Andrade’s [27] and Schmidtke and Heuer’s assumptions [35]. 

Finally, there are conflicting data on the role of attentional resources separate from memory processes to multiple contingency learning in incidental learning paradigms. Rowland and Shanks [13] found that learning of a secondary sequence in a dual-task SRT design was abolished under conditions of high perceptual load suggesting attentional limitations on learning of multiple contingencies, though there was evidence of learning across both tasks when perceptual load was low. However, in this study the secondary task constituted a statistically independent secondary sequence task, appearing at one of four locations above the visual array (four locations) for the primary sequence. The authors concluded that it remains to be established whether learning of multiple contingencies is incidental or explicit and whether findings reflect effect of secondary load on incidental or explicit learning, or a combination. Emberson, Conway and Christiansen [14] also proposed that directed attention is a prerequisite for incidental learning because they found no evidence of learning in any unattended information stream regardless of stimulus modality or presentation timing. However, it is generally accepted in incidental learning paradigms that a stimulus array must be attended too, even in cases where allocation of attentional resources is covert, for learning to occur [27]. In contrast, other work has shown that dual-motor sequences can be acquired without significant interference effects between tasks [36]. 

To conclude, incidental learning is a robust phenomenon when measured by single-task SRT tasks. Dual-task designs have been employed to investigate the putative contribution of attention and working memory to incidental and secondary task learning with the typical convention of adopting an auditory tone-counting task as the secondary task. One problem is that whilst a secondary auditory task will load attention and to some extent verbal working memory, it is unlikely to load visuospatial processes. Studies investigating the contribution of visual and verbal memory processes to incidental learning typically present secondary stimuli before, within (inter-trial stimulus) or after the primary SRT task stimuli [11,16,19,23,24,25], a convention that is also prevalent when a secondary auditory task is used, rather than present secondary stimuli synchronous with primary task stimuli. Findings generally show no effect of secondary tasks on memory although there is evidence from correlational studies that working memory processes may contribute to incidental learning. Other studies have developed novel dual-task designs that embedded and synchronised visual stimuli [10], or presented auditory stimuli that co-varied with sequence stimuli [17,28], and showed conflicting findings that may reflect perceptual modality of secondary task used or some other aspect of experimental design. Importantly, in these studies working memory tasks did not comprise the secondary load. In light of these mixed findings the contribution of explicit visual/verbal and visuospatial working memory processes to incidental SRT task learning, with secondary task stimuli embedded within and synchronised with the primary task, remains to be determined. 

The present study addressed the possible contribution of explicit working memory processes to incidental learning in a novel design comparing learning on a single- compared to dual-task SRT with verbal and visual memory tasks as secondary task stimuli [1,11]. In the present study a conventional SRT was replicated from Seger’s [1] study for the single-task learning condition and modified for dual-task conditions. Previous data show robust learning in healthy controls on the selected SRT task indicating that it reliably captures incidental learning [1,2,3,4,5]. Results of the present study should be comparable to other dual-task studies and may help resolve some of the conflicting findings. 

The present experiment had seven conditions; a single-SRT task, Verbal dual-task, Verbal-Spatial Above, Verbal-Spatial Below, Visual dual-task, Visual-Spatial Above and Visual-Spatial Below. In Visual and Verbal dual-task conditions secondary visual or verbal memory stimuli appeared at screen locations in the primary SRT in place of the stimulus circle for approximately half of stimulus trials. Thus, secondary memory task stimuli were embedded and synchronous with the primary SRT task stimuli simultaneously constituting a SRT trial requiring a RT response and target stimuli for the secondary memory task similar to the Jiménez and Méndez study [10]. In the Verbal-Spatial and Visual-Spatial Above conditions secondary memory task stimuli appeared at sequence locations in place of the SRT stimulus circle but were spatially decoupled from the sequence by appearing above the locus of the sequence array where the circle stimuli appeared (that were not replaced by icons/digits). Thus, secondary task stimuli were embedded and synchronous with the primary SRT (appearing in place of a circle and requiring a RT response and thus following the sequence) but were *spatially* displaced to evaluate the putative contribution of spatial processing to incidental learning separate from visual and verbal memory processes. Verbal-Spatial Below and Visual-Spatial Below conditions again presented secondary stimuli that were temporally embedded and synchronous with the primary task, but were spatially decoupled, appearing below the screen location where the stimulus circle would normally appear. Visual-Spatial and Verbal-Spatial Below conditions were thought likely to snare spatial attention more effectively than “Above” conditions because scanning below a horizontal visual array is more demanding than diverting attention above a horizontal visual array [37]. It was anticipated that secondary visual memory tasks should disrupt learning more than verbal tasks even though the verbal task was presented visually. Concomitantly, Visual-Spatial and Verbal-Spatial Above and Below conditions were expected to make increased demands on attention and memory processes compared to the Single- and Visual and Verbal Dual-tasks, because the participant had to divert attention above or below the central visual array where target circles appeared to process secondary icons/digits. Thus incidental and explicit learning score were expected to incrementally decrease from Single-, through Verbal-Dual, Verbal-Spatial A, Verbal-Spatial B, Visual-dual, Visual-Spatial A and Visual-Spatial B conditions if secondary task load made increased demands on memory/attentional processes across conditions and recruited the same resources required for incidental and explicit sequence learning. In the present study incidental sequence learning, explicit sequence learning and acquisition of the secondary learning/memory task proceed concurrently with primary and secondary tasks simultaneously recruiting attentional and memory processes representing a significant departure from conventional dual-task designs.

## 2. Experimental Section

### 2.1. Participants

Participants (*N* = 140, 70 male, 70 female, age *M* = 33, SD 12. 5) were recruited to the study with (*n* = 20) randomly assigned to one of seven conditions: Single-task, Verbal dual-, Verbal-Spatial Above, Verbal-Spatial Below, Visual dual-, Visual-Spatial Above and Visual-Spatial Below conditions. Age of participants was not significantly different across condition, *F*(6,133) = 1.26, *p* = 0.28 two-tailed. 

### 2.2. Single-Task Condition

A conventional SRT task [11] used in other work and replicated from Seger [1,2,3,4,5] was adapted for dual-task conditions with the canonical version of the task used to index sequence learning in the Single-Task condition. The task was programmed in Psyscope [38]. Participants completed a practice session to establish screen location/key press contingencies before beginning the task. Duration of practice was participant-determined but did not extend beyond the random block of 50 trials. In the learning phase participants were instructed to respond as quickly as possible to a target (1 cm white circle) appearing in a predetermined 10 trial sequence, A B C D B C B D B C, at one of four locations on the screen. The target was a 1 cm diameter closed white circle that could appear at one of four evenly spaced locations on a black background. The four screen locations were arranged horizontally and were unmarked. The two end circles were separated by approximately seven degrees of visual angle. On each trial one of the four circles appeared in its assigned screen location. The circles were programmed to disappear from the screen only when the appropriate key press was made. Circle screen locations corresponded to specific keys on the keyboard in the following way: when a circle appeared at the leftmost location key “v” was pressed; inner left location corresponded to key “b”, inner right location corresponded to key “n”, and far right location to key “m”. The sequence was a hybrid structure ABCDBCBDBC that contained unique elements (A, D) where each element has a unique following element and, ambiguous elements (B, C) where elements predict more than one other element [1]. The first trial of each block always began with the circle presentation appearing at a different location from the previous block. In this study two of five screen assignments used by Seger (1997) [1] were adopted for counterbalancing. In the first screen assignment, circles appeared with greater frequency at inner left and inner right screen positions. In the second screen assignment circles appeared with greater frequency at outer right and outer left screen positions. Both assignments also differed in the tendency of the sequence to skip locations, for example in one screen assignment the circle might appear at outer left, inner right, outer right locations skipping the inner left location, and in the other screen assignment the circle might appear at inner left, inner right, outer right, skipping the outer left location. This ensured that learning of the sequence was not dependent upon the frequency with which circles appeared at any given screen location. The screen assignments and sequences used in the SRT task are presented below and are replicated from Seger [1] see also Stadler for greater detail on the statistical structure of the sequence used [39]. Stimulus screen location frequency counterbalancing was used across all conditions, with participants randomly assigned to assignment one or two (see Table 1).

**Table 1:** Counterbalancing of screen locations for the SRT task.

Sequence	Screen location assignments for the sequence									
	A	B	C	D	B	C	B	D	B	C
**Assignment 1**	1	3	2	4	3	2	3	4	3	2 *
**Assignment 2**	2	1	4	3	1	4	1	3	1	4

* 1–4 corresponds to furthest-left furthest-right screen locations respectively.

For random blocks, random strings of ten stimulus locations were formulated that shared the probabilistic features of the sequence but did not mirror the sequence. In this way it was ensured that circles in random blocks appeared at each location with the same frequency as circles in the sequence blocks although they did not follow the sequence. This first order approximation of the sequence required several rules that a truly random pattern would not follow. For example, it was important that a circle did not appear twice in succession at the same screen location and also that the random sequence did not follow a pattern of 4321 or 1234. If either of these events had occurred it would have meant that the random trials were clearly distinguishable from the sequence and therefore more likely that participants would have explicitly detected that sequence blocks followed a pattern. Programming of random blocks as a first order approximation of the sequence ensured that differences between sequence and random blocks at test did not result from mere learning of first order frequency information [40]. In accordance with these principles five first order random presentations, consisting of ten trials each, were formulated for each of the random blocks (one at the beginning of the task and two at test stage). In dual-task designs memory stimuli also followed the frequency rule and did not appear consecutively at the same stimulus location. 

The circle remained on the screen until the correct key press was made and reaction time responses (RT’s) to each trial were recorded. In random blocks the stimulus circle appeared with the same frequency at screen locations as sequence blocks but did not follow a sequence. The response-stimulus interval was 200 ms. The learning phase consisted of seven blocks of 50 trials comprising an initial random block to discourage participants from explicitly assuming that circles followed a pattern at the outset of the experiment, followed by six sequence (learning) blocks. Test phase comprised one sequence block flanked by two random blocks and followed immediately after the learning phase without warning to participants. Self-determined rest breaks appeared after each block of 50 trials. 

Each of the three test blocks (two random and one sequence block) produced 50 reaction time values, divisible as five repeats of ten trials. Median RT’s for each of the five repeats of ten trials were calculated. The five medians for each block were combined to produce three means, one sequence mean and one mean for each random block. The two random block means were combined to produce a single mean. The sequence mean was subtracted from the random mean to provide a single learning score for each participant. After the task participants completed the explicit knowledge questionnaire used in earlier studies [1,2,3,4,5]. 

*Explicit measure*: The explicit awareness measure established whether participants were explicitly aware of the presence of the sequence, whether they could describe the sequence and whether they had determined the sequence length. Instructions were as follows.

*Please answer these questions in the order given.*

*“In this experiment you were presented with circles. In reality there was a pattern to the occurrence of the circles. Of course the purpose of the experiment wasn’t to look for patterns, and often participants do better on these types of experiment if they are not aware of any pattern. However, we are curious about the degree to which people notice the presence of a pattern. Please rate how aware you were of any pattern by making a mark on the scale below.”*

The first question of the explicit measure asked participants to rate how certain they were of the presence of a pattern on a 7-point unnumbered visual analogue scale (0–6). At the far left of the scale the sentence, “*I did not even suspect there was a pattern*”was printed. At the far right, “*I was completely certain there was a pattern*”was printed. The second question asked participants to describe any pattern noticed. This section had to be completed before viewing the final two questions presented on the following page. Question three asked participants how sure they were that the sequence consisted of ten locations. The fourth question asked participants to rate how sure they were that the sequence consisted of 12 or so positions. Third and fourth question responses were measured by a 7-point (0–6) visual analogue scale, and the sentence, “*I think it is very unlikely that is the pattern*” appeared at the far left of the scale and “*I think it is very likely that is the pattern*” at the far right. 

The second question of the explicit measure required that participants recalled and described any sequence that they were aware of, the third and fourth questions required that participants recognise the correct sequence length from two options. The explicit measure therefore comprised questions that drew on free recall and questions that measured participants’ recognition of the sequence length. Scoring of the explicit measure corresponded to Seger’s [1] original scoring template, whereby each participants’ score comprised their ratings on question one (awareness of sequence), question three (recognition that the sequence comprised ten trials) combined with their score on question two (description of the sequence). Ability to describe the sequence was rated on a six-point scale (0–5, question two). Participants were given a score of nought if they mentioned a false feature and five if they provided a complete correct description. They were given a score of one if they mentioned a sequence without further details; two if they mentioned the base frequencies; three if they identified a short run of length three or four; and four if they showed almost complete knowledge by identifying a run of five positions or more. Scores on this section of the explicit measure were doubled before being added to the awareness and recognition ratings. Following previous studies [1,2,3,4,5], a score of sixteen or over constituted evidence that the participant was explicitly aware of the sequence. 

Learning phase (acquisition), test phase (random, sequence, random blocks with no secondary stimuli present for dual-tasks) and the explicit knowledge measure were the same across all conditions. Each participant completed a practice session to become familiar with key/location contingencies prior to beginning the experiment. Participants were instructed to press corresponding keys as quickly as possible when stimuli appeared at one of four screen locations. 

### 2.3. Verbal Dual-Condition

Single digit numbers were selected to appear at approximately half of screen locations in place of stimulus circles for the seven acquisition blocks prior to testing phase (excluding 0 which might have been confused with a stimulus circle). Participants were instructed to count the number of digits appearing and key in their response at the end of each block. Each block had a different digit stimulus (*i.e.*, block 1 = digit 2 appeared, block 2 = digit 8 appeared, *etc.*) and the frequency and location of digit presentations varied across blocks (but always appeared at least once at each stimulus circle location) to deter guessing, but approximated around half of sequence stimulus trials and varied between 22 and 28 trials, a spread of three digits either side of the mean of 25 trials (*i.e.*, 22 for block 1, 24 for block 2, *etc.*) with the 7 possible frequencies of digit presentation used once across the 7 acquisition blocks. Ordering of digit frequency over blocks was counterbalanced across participants. This was important to counteract possible order effects and also because there is some suggestion that frequency detection can lead to explicit detection of an implicit sequence (see Single-task description and Stadler [39], and Shanks and St. John [40]). At the beginning of the experiment participants were instructed to respond to stimuli as quickly as possible whilst pressing the appropriate key and to count the number of digits appearing in each block. At the end of each block participants were instructed to key in the number of digits they had counted. Test procedure was the same as the Single-task condition. Learning scores for the primary task, accuracy scores for the secondary task and explicit measure scores were calculated for each participant. 

### 2.4. Visual Dual-Condition

**Figure 1:**
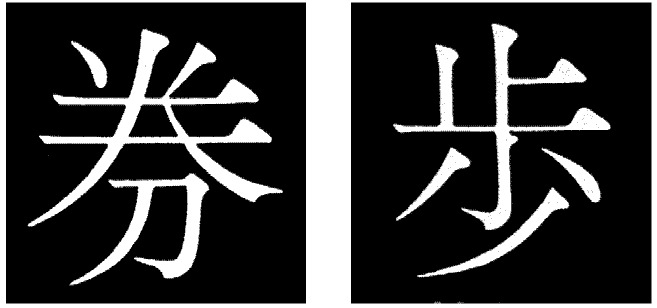
Japanese icons appearing in place of circle stimuli for approximately 50% trials in a SRT task acquisition blocks (*n* = 7) for Visual and Visual-Spatial dual-task conditions.

Japanese icons were selected as visual stimuli embedded within the primary SRT task to recruit visual working memory processes. Icons were selected to prohibit participants from verbally recoding visual information if the primary task made high demands on visual working memory. For example, many familiar objects presented visually can be verbally recoded (*i.e.*, green chair with four legs). The selection of Japanese icons was intended to deter any verbal recoding of visual information due to the abstract and visually complex nature of the icons. Participants were instructed to press the corresponding key as quickly as possible whether a circle or an icon appeared at one of the four target screen locations. Seven pairs of Japanese icons were selected that shared similar features based on piloting data (see Figure 1). Each pair was assigned to one of the seven acquisition blocks with one of the icons appearing in place of circle stimuli at approximately half of stimulus trials that varied between 22 and 28 trials in accordance with Verbal conditions. Both icons (target and foil) were presented at the end of each block. Since icon pairs selected for each block were matched on several dimensions participants had to attend too them, and discriminate between them in order to accurately identify the presented target icon from the foil icon at the end of each block. For three of the seven blocks at acquisition neither icon presented at the end of the block had been featured during stimulus trials to deter accuracy scores that merely reflected guessing. Participants were instructed to attend to the icon appearing in each block and at the end of the block determine whether they had seen the presented icon before by distinguishing between two presented icons (target and foil) and pressing “y” for “yes” or “n” for “no”. For three of the acquisition blocks the correct response to both target and foil icons was “no” because neither had appeared in the previous block. Test procedure was the same as the Single-task condition. Learning scores for the primary task, accuracy scores for the secondary task and explicit measure scores were calculated for each participant. 

### 2.5. Verbal-Spatial above and Verbal-Spatial below Conditions

Verbal-Spatial A and B dual-task conditions were the same as the Verbal dual-task condition except that single digits temporally embedded within the sequence appeared at 1.7 degree of visual angle above (Verbal-Spatial A) or below (Verbal-Spatial B) the horizontal array where the stimulus circle typically appeared (spatially asynchronous). Frequency and location of number presentations varied across blocks to deter guessing but approximated around half of the stimulus trials as with the Verbal condition. Participant instructions were the same as the Verbal condition; test procedure and explicit measure were the same as the other conditions. Incidental and explicit learning score, and accuracy data were calculated for each participant. 

### 2.6 Visual-Spatial above and Visual-Spatial below Conditions

The experimental design was the same as the visual-dual task condition with the only difference that stimuli were spatially asynchronous with SRT circle stimuli appearing instead at 1.7 degree of visual angle above (Visual-Spatial A) or below (Visual-Spatial B) the location where circles typically appeared. At the end of each block participants were presented with two icons and asked to identify if they recognised one of the stimulus items from the previous block. Incidental learning score, accuracy data and explicit earning scores were calculated for each participant. It was anticipated that there would be a proportionate diminution in learning score and explicit awareness/learning score on the basis of condition if secondary load recruited the same resources required for incidental SRT and explicit sequence learning. 

It was expected that Verbal-Spatial Above and Visual-Spatial Above conditions would make fewer demands on attentional resources than Verbal-Spatial Below and Visual-Spatial Below, with Visual-Spatial Below expected to constitute the most difficult dual-task condition, because it is easier to scan above a visual horizontal array (divert attention above an array) than below and both primary and secondary tasks made demands on visual memory resources [37]. The expression of incidental learning and explicit sequence learning was expected to diminish from Single-task through, Verbal-dual, Verbal-Spatial A, Verbal-Spatial B, Visual-dual, Visual-Spatial A and Visual-Spatial B conditions if secondary task load made increased demands on memory/attentional processes across condition, and recruited the same resources required for expression of incidental and explicit sequence learning.

## 3. Results

Mean incidental learning scores, explicit learning scores and percent accuracy scores were calculated for each condition (see Table 2). Percentage accuracy scores, rather than overall accuracy score, were used in subsequent analyses because this value was considered a more accurate and fine-grained representation of participants’ performance than total accuracy score. Mean percent accuracy scores were calculated for each participant for each digit value response in Verbal/Verbal-Spatial A and B conditions and for mean overall accuracy in the Visual/Visual-Spatial A and B conditions as follows: Verbal experiment—where “a” signifies participant’s response for digits counted across block (23 in this example) and “b” represents the correct answer (26), the participant’s response is 88% accurate:

a(23) ÷ b(26) × 100 = 88% accurate

In circumstances where there were false positives (the correct answer was 26 and the participant reported 27), the false positive (one value) was deducted from the correct total counted (26 in this example) to produce an adjusted response of 25. Percentages were combined for the seven acquisition blocks and averaged to produce a mean percentage accuracy score for each participant across blocks. The same procedure was followed for the Visual experiment where “a” represented the number of correct yes/no responses and “b” represented total possible number of correct responses (*n* = 7). 

**Table 2:** Descriptive data for incidental learning, explicit learning and percentage accuracy scores × condition.

Condition	Incidental learning scoreMean (SD)	Explicit learning scoreMean (SD)	Digits counted: % accuracyMean (SD)	Icons identified: % accuracyMean (SD)
**Single-task**	83.4 (49.6)	13.8 (4.4)		
**Verbal dual-task**	64.9 (33.8)	7.5 (3.8)	94.7 (4.9)	
**Verbal-Spatial A**	77.3 (53.1)	4.5 (2.3)	93.7 (7.7)	
**Verbal-Spatial B**	62.1 (40.8)	5.6 (3.1)	89.7 (11.8)	
**Visual dual-task**	73.5 (36.6)	8.3 (3.3)		85.6 (11.4)
**Visual-Spatial A**	71.9 (39.7)	3.8 (2.3)		84.9 (15.7)
**Visual-Spatial B**	64.8 (26.1)	3.9 (1.8)		82. 8 (15.8)
**Total group**	71.1 (39.9)	6.7 (3.0)	92.7 (8.1)	84.4 (13.5)

Descriptive data presented in Table 2 show that incidental learning scores were similar across conditions and did not appear to incrementally diminish from Single- to Visual-Spatial B conditions as predicted on the basis of increasing demands on shared processes across primary SRT and secondary tasks. The Single-task condition mean incidental learning score was greater than incidental learning mean scores across conditions but standard deviations were also large across groups. Verbal-Spatial A condition had a relatively greater mean incidental learning score than other dual-task conditions, but the standard deviation value was also greater for this compared to other dual-task conditions arguably indicating greater variability across scores for Verbal-Spatial A compared to other conditions. A One-Way ANOVA conducted with learning score as the dependent variable and condition the between-groups factor showed that incidental learning score did not differ as an effect of condition *F*(6,133) = 0.71, *p* = 0.64 two-tailed. There was no significant linear trend for incidental learning score *t*(1,133) = 0.82, *p* = 0.36 two-tailed, indicating that learning score did not incrementally diminish from the Single-task condition to Verbal dual-, Verbal-Spatial A, Verbal-Spatial B, Visual dual-, Visual-Spatial A and Visual-Spatial B conditions as expected if primary SRT and secondary memory task performance depended on shared cognitive resources. 

Explicit learning/awareness scores presented in Table 2 indicated a general trend of incremental diminution as an effect of condition as predicted. Results of a One-Way ANOVA showed that explicit scores differed significantly as an effect of condition *F*(6,133) = 25.7, *p* ≤ 0.01 two-tailed. Results of Tukey’s *post-hoc* comparisons (corrected for multiple analyses) showed that explicit scores in the Single-Task (*M* = 13.8, 95% CI [11.7, 15.9]) condition differed significantly from Verbal dual condition scores (*M* = 7.5, 95% CI [5.7, 9.3]) *p* ≤ 0.01, Visual dual condition scores (*M* = 8.3, 95% CI [6.7, 9.8]) *p* ≤ 0.01, Verbal-Spatial A condition scores (*M* = 4.5, 95% CI [3.3, 5.5]) *p* = 0.00, Verbal-Spatial B condition scores (*M* = 5.6, 95% CI [4.1, 7.1]) *p* ≤ 0.01, Visual-Spatial A condition scores (*M* = 3.8, 95% CI [2.7, 4.9]) *p* ≤ 0.01, and Visual-Spatial B condition scores (*M* = 4.1, 95% CI [3.3, 4.9]) *p* ≤ 0.01. In sum, Single-task explicit scores differed significantly from the explicit mean scores of each other experimental condition. There were also differences between explicit learning/awareness scores for dual-task conditions. For the Verbal dual-task condition, mean explicit score differed significantly from Verbal-Spatial A *p* = 0.03, Visual-Spatial A, *p* ≤ 0.01, and Visual-Spatial B conditions, *p* = 0.01, but not for the Visual dual-task, *p* = 0.99, or Verbal-Spatial B conditions, *p* = 0.44. Similarly, the Visual dual-task explicit scores differed significantly from Verbal-Spatial A, *p* ≤ 0.01, Visual-Spatial A, *p* ≤ 0.01 and Visual-Spatial B conditions, *p* = 0.01, but not Verbal-Spatial B, *p* = 0.10. There were no other differences. These findings indicate that spatially asynchronous conditions (with the exception of Verbal-Spatial B) made greater demands on resources required for expression of explicit sequence learning than Visual and Verbal dual-task conditions comprised of spatially embedded and temporally synchronous secondary stimuli. The lack of difference between Visual/Verbal dual-task and Verbal-Spatial B conditions might be due to participants’ attending less to secondary task stimuli; descriptive percentage accuracy scores indicated that participants were least accurate in the Verbal-Spatial B condition of all Verbal conditions. Hence, there may have been some trade-off between performance accuracy, incidental learning and explicit learning in this condition. Descriptive data also indicated that Visual dual-task conditions were generally more difficult than Verbal conditions based on mean accuracy scores, which is not surprising since the SRT task is primarily a visuospatial task. 

Linear contrast analyses were conducted to investigate whether the suggested trend across conditions in explicit awareness descriptive data was a significant effect of secondary task performance on explicit learning. Contrast data were input in the same order as for other analyses to establish whether a significant linear trend, not seen in incidental learning data, was present in explicit learning data supporting a proportionate effect of condition on cognitive resources. Analyses revealed a significant trend for explicit scores to incrementally decrease from the Single- to Verbal dual-, Verbal-Spatial A, Verbal-Spatial B, Visual dual-, Visual-Spatial A and Visual-Spatial B conditions, *t*(1,133) = 6.77, *p* ≤ 0.01 two-tailed. However, despite the significant trend descriptive data showed similar explicit mean learning scores for Visual- and Verbal-dual tasks, suggesting that these conditions were more similar in terms of secondary task effects on explicit learning than Verbal- and Visual dual-tasks were to Verbal- and Visual-Spatial conditions. 

Overall group means presented in Table 2 show that participants were 84.4% accurate overall for Visual conditions and 92.7% accurate overall for Verbal conditions. Results of a One-way ANOVA showed no effect of condition on percentage accuracy of secondary task performance for Verbal conditions, *F*(2,57) = 1.9, *p* = 0.15 two-tailed. Similarly, there was no effect of condition on percentage accuracy of secondary task performance, *F*(2,57) = 0.44, *p* = 0.64 two-tailed for Visual conditions. However, descriptive data indicated that performance accuracy for the secondary task was greatest for the Verbal dual-task condition compared to other conditions and that performance accuracy for the Visual-Spatial B condition was least accurate, although incidental learning scores were similar for both conditions. These data support the assumption that Visual-Spatial B condition should make most demands on resources required for explicit learning and secondary memory task performance during concurrent SRT learning compared to other conditions. Consequently, accuracy data for Visual and Verbal dual-task conditions were compared in a One-Way ANOVA with linear contrasts to establish whether accuracy data followed a significant trend corresponding to explicit data potentially revealing an explicit learning/accuracy trade-off. Analyses revealed that condition had a significant effect on accuracy *F*(5,114) = 3.37, *p* ≤ 0.01 two-tailed. *Post hoc* analyses corrected for multiple comparisons revealed that Verbal dual-task condition accuracy score (*M* = 94.7, 95% CI [92.4, 97.0]) was significantly greater than Visual-Spatial B accuracy score (*M* = 82.8, 95% CI [75.4, 90.2]), *p* = 0.02 two-tailed, and Verbal-Spatial A condition accuracy scores (*M* = 93.7, 95% CI [90.1, 97.93]) condition accuracy scores were also marginally significantly greater than Visual-Spatial B condition scores, *p* = 0.05 two-tailed. Notably, percentage accuracy scores significantly and proportionately decreased from Verbal dual-, to Verbal-Spatial A, Verbal-Spatial B, Visual dual-, Visual-Spatial A and Visual-Spatial B conditions *t*(1,114) = 16.2, *p* ≤ 0.01 two tailed corresponding to results of analyses for explicit data across conditions. 

Overall results of analyses showed no significant effect of secondary task load on expression of incidental learning, but significant effects of condition on expression of explicit learning and percentage accuracy scores for secondary working memory tasks that followed a similarly significant linear trend across conditions. 

## 4. Discussion

In the present experiment there was one Single- and six dual-task conditions designed to increase in difficulty from Single-task through Verbal dual-, Verbal-Spatial A, Verbal-Spatial B, Visual dual-, Visual-Spatial A and Visual-Spatial B condition order based on assumed attentional demands of secondary tasks. For Verbal and Visual conditions secondary visual icons and verbal digits were synchronous with the primary SRT task by constituting a sequence trial in place of a stimulus circle for approximately half of trials across acquisition blocks and also comprising target stimuli for visual/verbal secondary working memory tasks. However, in Visual-Spatial and Verbal-Spatial conditions icons and digits were temporally synchronous with the primary SRT task but were spatially asynchronous (decoupled) from the central sequence circle stimulus array. It was anticipated that Visual dual-task conditions would recruit resources required for expression of SRT and explicit sequence task learning more than Verbal dual-tasks because the SRT task is primarily a visuospatial motor task. It was also anticipated that secondary spatial conditions would have a greater effect on sequence learning than Visual and Verbal conditions, because spatial displacement of secondary task stimuli necessitated gaze shift away from the central SRT stimulus circle array, with Visual-Spatial B making the greatest demands on memory/attentional processes compared to other conditions during concurrent SRT task learning. 

Results showed that the expression of incidental learning was not different as an effect of condition. Single-condition mean incidental learning score was greater than for dual-task conditions, but standard deviations were also relatively large across conditions. Analyses revealed that incidental learning scores did not follow an anticipated significant linear trend across conditions, and there was no evidence of a proportionate diminution in learning score through Single- to Visual-Spatial B as anticipated. Although there were only twenty participants in each condition, this task has been shown to be sensitive to differences in amount of learning in small groups across studies (2–5), and the overall cohort was relatively large making it unlikely that null effects represent small sample size, particularly since there were significant differences for other variables as an effect of condition. In contrast, explicit sequence learning/awareness scores were significantly greater for the single-task compared to dual-task conditions with explicit learning means following a significant linear trend from the Single- through to Visual-Spatial B condition, as anticipated if secondary load made increasing demands on the expression of explicit learning as an effect of condition. Interestingly, secondary task accuracy scores also followed a similar significant linear trend across conditions from Single- through to Visual-Spatial B conditions corresponding to the pattern of explicit data. These results indicate an accuracy/learning trade-off for the expression of explicit sequence learning in the present experiment that was not present for incidental sequence learning data. Single-task explicit scores differed significantly from the explicit mean scores of each other experimental condition. There were also differences between explicit learning/awareness scores for dual-task conditions. Results indicated that spatially asynchronous conditions (with the exception of Verbal-Spatial B) made greater demands on expression of explicit sequence learning than Visual and Verbal dual-task conditions comprised of spatially embedded and temporally synchronous secondary stimuli.

Present findings concur with other studies showing minimal effect of working memory secondary tasks on incidental learning, although there is broad variability across study design [18,28,29,36]. Gobel, Sanchez and Reber recently found that explicit scores dissociated from overall sequence learning ability in two experiments, similar to present findings, and concluded that incidental sequence learning proceeded implicitly [22]. Other findings have shown that two concurrent implicit tasks can be acquired without competition for cognitive resources [17]. 

Previous studies have typically presented secondary memory or tone counting tasks before, after or between incidental sequence stimulus trials [11,14,15,16,21,22,23], with only a few reported exceptions [11,17]. In this way stimuli can be considered “embedded” within the sequence but asynchronous with the sequence. That is, separate responses are required for primary and secondary task trials, and/or secondary stimuli do not simultaneously comprise a component of the primary sequence task. Several findings have revealed that pairing a SRT task with a secondary tone-counting task results in diminished incidental sequence learning [11,13,41]. However, reasons for this remain unclear though it has been hypothesised that secondary task trials might impact on incidental learning by simultaneously drawing on a limited capacity attentional resource [12,13,19,26], interfering with the automatic maintenance of sequential elements in working memory [24], producing a processing bottleneck because separate motor responses must be generated for each task stimulus [21], or by disrupting integrated elements of the sequence [39]. Present findings show that secondary visual, verbal, visual spatial and verbal spatial conditions did not disrupt the expression of incidental learning inconsistent with *attentional* and *working memory interference* hypotheses, although these theories provide plausible explanations for dual-task effects on explicit learning and secondary task performance accuracy. 

The reported significant incremental decrease in explicit learning and performance accuracy scores across conditions in the present study shown by corresponding linear contrast data results for explicit learning and accuracy data, suggests that processes required for deliberative encoding (participants were instructed to memorize or count secondary stimuli), maintenance, recollection and recall of stimulus information are crucial to the expression of explicit sequence learning but less important for incidental learning. Jiménez and Vázquez similarly found that a SRT and contextual cueing task could be acquired concurrently without diminution of learning on either task so long as learning on both tasks remained incidental [17]. Inter-task interference only occurred when sequence information became explicit resulting in diminution of the expression of contextual cue learning not SRT task learning. Present findings also indicate an explicit learning/secondary memory task trade-off supporting the assumption that explicit learning depends upon finite resources and that incidental learning may proceed independently and relatively automatically. 

Results of the current study also concur with data showing that when a single motor response is required for primary and secondary stimuli (because secondary stimuli are embedded within and synchronous with the primary task), incidental learning is unaffected by the secondary task [10,17]. Considered together these findings provide indirect support for the processing *bottleneck* theory of dual-task effects. Schumacher and Schwarb demonstrated successful learning under various dual-task conditions but found that sequence learning was diminished when parallel responses were required for both tasks [21]. In contrast, sequence learning proceeded normally under serial response selection conditions. The authors argued that findings conflict with attentional [12,13,19,26], automatic [24], dual-learning mechanism [23], and integrative [35,39] hypotheses, proposing instead that findings occurred due to overlap of central processes involved in successful dual-task performance. However the processing bottleneck theory could arguably be accused of suffering from similar limitations to other attentional and/or limited capacity resource explanations, because the theory is conceptually similar to these approaches. Cheyne, Ferrari and Cheyne [42] have suggested an alternative explanation for effects of parallel response selection on expression of incidental learning. They used MEG (magnetoencephalography) techniques to track the time course of neural activity in frontal and motor regions thought to reflect conscious and controlled inhibition of pre-potent responses and selection of alternate responses to an infrequent switch cue. The authors concluded that automatic (implicit/incidental) and controlled processes engage in parallel during rapid motor response tasks, and that strength and timing of these processes potentially underlies optimal task performance. Gobel, Sanchez and Reber similarly found that when precise timing is necessary for task performance, sequence learning depends on an integrated representation of sequential action and interaction timing information, indicating the importance of both timing (temporal information) and stimulus integration [22]. Other studies adopting similar embedded/synchronous dual-task designs to the one presented here have shown that when tones co-vary with sequence locations the disruptive effects of secondary auditory tone counting on expression of incidental learning decreases [17,35], again suggesting that stimulus integration abolished dual-task effects on incidental learning. However, in support of the “bottleneck” hypothesis, McBride *et al.* reviewed evidence showing significant overlap between brain regions active during consciously and unconsciously triggered action control [43]. These findings provide a potential neural analogue to Schumacher and Schwarb’s notion of central cognitive resource overlap during parallel motor response selection in dual-SRT tasks [21]. 

Importantly, spatially asynchronous stimuli similarly did not impede the expression of incidental learning in the present study. This may have been because secondary stimuli were embedded within and *temporally* synchronous with the primary task across conditions regardless of spatial synchronicity or asynchronicity, simultaneously constituting a sequence element *and* secondary task target. However, contrasting with this explanation Heuer and Schmidtke found that embedded but *temporally* asynchronous visual and verbal secondary tasks also did not disrupt incidental learning, although sequential presentation of primary and secondary task stimuli may have enabled participants to switch attention between stimulus items in their study [25]. Interestingly, a go/no go task requiring a foot pedal response interfered with primary sequence learning, again supporting the notion that conflicting parallel motor responses diminish SRT task learning [21]. Present findings indicate that expression of incidental sequence learning is robust to spatial displacement of sequence trials, even when those sequence elements require visuospatial processing to meet secondary task requirements. Thus, spatially displaced secondary targets do not disrupt the expression of incidental sequence learning so long as primary and secondary stimuli are temporally synchronous requiring only a single motor response to both stimulus targets. 

On the basis of current and previous findings, degree of synchronicity of primary and secondary task stimuli seems an important determinant of whether dual-task effects are seen when there are no competing motor responses for task completion. Results reported here suggest that timing/temporal ordering of learning trials may be a crucial, and likely automatic process contributing to incidental motor learning above the contribution of memory and attentional processes. Future work might use embedded and synchronous stimuli but specify a different or multiple competing response(s) to some element of secondary stimuli to explore this possibility further. Additionally, it is likely that several other boundary conditions govern how attention is allocated to particular task-stimuli and the capacity restraints of these resources. Task-relevance and intentional stance may be important factors, although Jiang and Leung showed that even task-irrelevant stimuli were “attended to” evidenced by facilitation effects on visual search when previously irrelevant cues became relevant after a switch [44]. Their findings suggest that attentional filtering occurs late in the learning process. In contrast, Schmidtke and Heuer found that when secondary tones were presented without instruction the secondary sequence was only minimally learned indicating that mechanisms of incidental learning are not wholly non-selective [35]. Similarly Knee, Thomasen, Ashe and Willingham [45] investigated the possibility that explicit sequence learning depends upon acquisition of stimulus locations, and incidental learning depends upon acquisition of motor sequences. This hypothesis goes some way to explain current findings of linear contrasts that visuospatially asynchronous conditions were more difficult than synchronous conditions, and had greater impact on explicit sequence learning. Knee *et al.* [45] have proposed that explicit learning in the SRT task is visuospatial in nature, whereas incidental learning is not. One possible way to provide further support for this explanation might be to modify the current experiment to include motor sequence and visuospatial sequence transfer blocks.

## 5. Conclusions

The present study design is unique in that secondary tasks were synchronous and spatially asynchronous with the primary SRT task depending upon condition, tapping modality specific visual, verbal and spatial memory processes. Findings support growing evidence that secondary attentional/memory tasks do not diminish expression of incidental learning [15,16,17,19,24], when secondary task stimuli are embedded and temporally synchronous and spatially asynchronous with the primary task. Ultimately, more research is needed to distinguish between competing theories. Nevertheless, the present design offers a useful set of conditions under which interfering effects of dual-task processing on expression of incidental and explicit learning can be usefully studied. In response to the question posed in the title of this article, conscious rules regarding limited capacity controlled attentional and memory resources do not seem to apply to expression of incidental learning on the basis of present and other findings, but are crucial for the expression of explicit sequence learning. In contrast stimulus temporal synchronicity and motor response selection mechanisms are plausibly fundamental to incidental learning. During acquisition of incidental sequence learning the key press response-to-stimulus represents an elemental motor programme (it cannot be broken down into smaller constituent actions) whereas the motor response to sequences depends upon synthesis of elemental programmes to represent the sequence, similar to playing a piece of music. Elemental motor patterns are likely encoded differently to complex patterns, which will have inherent variability (a ball will never be hit with exactly the same force, direction or trajectory) to allow for subtle compensatory adjustments to achieve the target/goal behaviour [46]. So explicit components of incidental learning might include perceptual encoding as Knee *et al*. have proposed [45], and also motor response selection/updating as Schumacher and Schwarb have suggested [21]. Thus, in incidental learning disruption of the elemental response-to-stimulus motor programme via introduction of inter-trial stimuli may likely affect how this motor pattern is neurally represented—relying more on mechanisms governing explicit sequence learning to incorporate motor adaptation and thus explaining some of the contrasting findings. Current findings suggest that temporal synchronicity is key to the expression of incidental learning, robust to interference effects of secondary Visual, Verbal, Visual-Spatial and Verbal-Spatial tasks, and limited capacity visual, verbal and spatial working memory processes are key to the expression of explicit sequence learning. The extent to which temporal coherence detection and motor response selection depend on conscious or non-conscious processes under varying conditions of attentional load remains to be determined, and future work should also evaluate whether models of incidental learning are neurally viable relative to what is now known about neural substrates of motor programmes. 
